# Why are Women With Polycystic Ovary Syndrome at Increased Risk of Depression? Exploring the Etiological Maze

**DOI:** 10.7759/cureus.13489

**Published:** 2021-02-22

**Authors:** Anupa Gnawali, Viral Patel, Alejandrina Cuello-Ramírez, Anoud S Al kaabi, Asfa Noor, Mohammed Y Rashid, Shereen Henin, Jihan A Mostafa

**Affiliations:** 1 Family Medicine, California Institute of Behavioral Neurosciences & Psychology, Fairfield, USA; 2 Research, California Institute of Behavioral Neurosciences & Psychology, Fairfield, USA; 3 Internal Medicine, California Institute of Behavioral Neurosciences & Psychology, Fairfield, USA; 4 Neonatology, California Institute of Behavioral Neurosciences & Psychology, Fairfield, USA; 5 General Surgery, California Institute of Behavioral Neurosciences & Psychology, Fairfield, USA; 6 Internal Medicine, Pediatrics, California Institute of Behavioral Neurosciences & Psychology, Fairfield, USA; 7 Psychiatry, California Institute of Behavioral Neurosciences & Psychology, Fairfield, USA

**Keywords:** insulin resistance, hyperandrogenism, menstrual irregularities, polycystic ovary syndrome (pcos), depression

## Abstract

Polycystic ovary syndrome (PCOS) is a complex and common multisystemic disorder. Women with PCOS have up to eight times higher prevalence of depression than control groups. This paper aims to explore the underlying risk factors for developing depression in this high-risk group.

Studies indicate an interplay of multiple mechanisms that place women with PCOS at an increased risk for depression. The pathophysiology thought to play a role includes disturbances in the endocrine axes and changes to the metabolic pathway. The risk of depression is independently linked to insulin resistance and obesity in this population. However, rates of depression were still higher than control groups when accounting for these variables, demonstrating that they are not the only mechanism causing depression. The clinical manifestations of hyperandrogenism and menstrual abnormalities may compound negative views and lower self-esteem and negatively impact mood. Many of these women also struggle with infertility, and due to the added external pressures like societal beliefs and culture, they may be further negatively impacted and worsen their depressive symptoms.

The prevalence of depression in women with PCOS is high; thus, this paper highlights the essential understanding of the underlying mechanisms at play. This is to better aid in addressing the fundamental cause of depression in this high-risk group effectively.

## Introduction and background

Polycystic ovary syndrome (PCOS) is a common and complex multisystemic disorder [1]. The women most severely impacted by PCOS are in their reproductive years. There is a rising prevalence of PCOS ranging between 8% and 13%, depending on the population studied and definitions used [2,3]. Depression significantly impacts the global burden of the disease, and it is the most common cause of disability worldwide [4]. Women with PCOS have a three to eight times higher prevalence of depression than control groups [5], emphasizing the importance of depression in patients with PCOS. The rising concern of depression has been recognized, and international guidelines now recommend screening depression among all women with PCOS at the time of diagnosis [6].

The diagnostic triad for PCOS comprises ovulatory dysfunction, hyperandrogenism, and polycystic ovaries on ultrasound [6]. The ovulatory dysfunction can present as oligomenorrhea or anovulation. These menstrual irregularities are associated with a poor feminine identity [7,8]. It is compounded by hyperandrogenism that can manifest biochemically or clinically (hirsutism and acne). These clinical features can lower self-esteem and have a further negative effect on mood [7-9]. Cinar et al. explored the link between androgens and mood disorders and concluded that it is not independently associated with depression [9].

Metabolic dysfunction in the form of insulin resistance and obesity is present in 60% of women with PCOS [10]. One of the significant risk factors for developing depression in the general population is obesity, which is also present in women with PCOS [11,12]. However, studies show an increase in the prevalence of depression in women with PCOS, even when controlling body mass index (BMI), suggesting that obesity is not the only mechanism leading to depression in these women [5,10]. Insulin resistance is independently and strongly associated with depression in women with PCOS and may play a physiological role. Even though insulin resistance has been linked with depression, the exact causal relationship is still unknown. Impaired insulin signaling may alter mood; alternatively, low mood might cause behavioral or central mechanisms that lead to insulin resistance [13].

Another feature of PCOS is infertility, which has been correlated to negative psychological stress and decreases the affected women’s quality of life. Reduced sexual satisfaction and self-worth were possible contributory factors. However, infertility and psychological stress are mostly dependent on ethnic background, religious beliefs, and personal desire for children [1,14,15]. Tan et al. concluded in a study in China that infertility was not a primary determinant of psychological problems [15]. In contrast, Behboodi Moghadam et al. determined that for Iranian women infertility was perceived as a significant concern due to the social pressures of having children [1].

**Figure 1:**
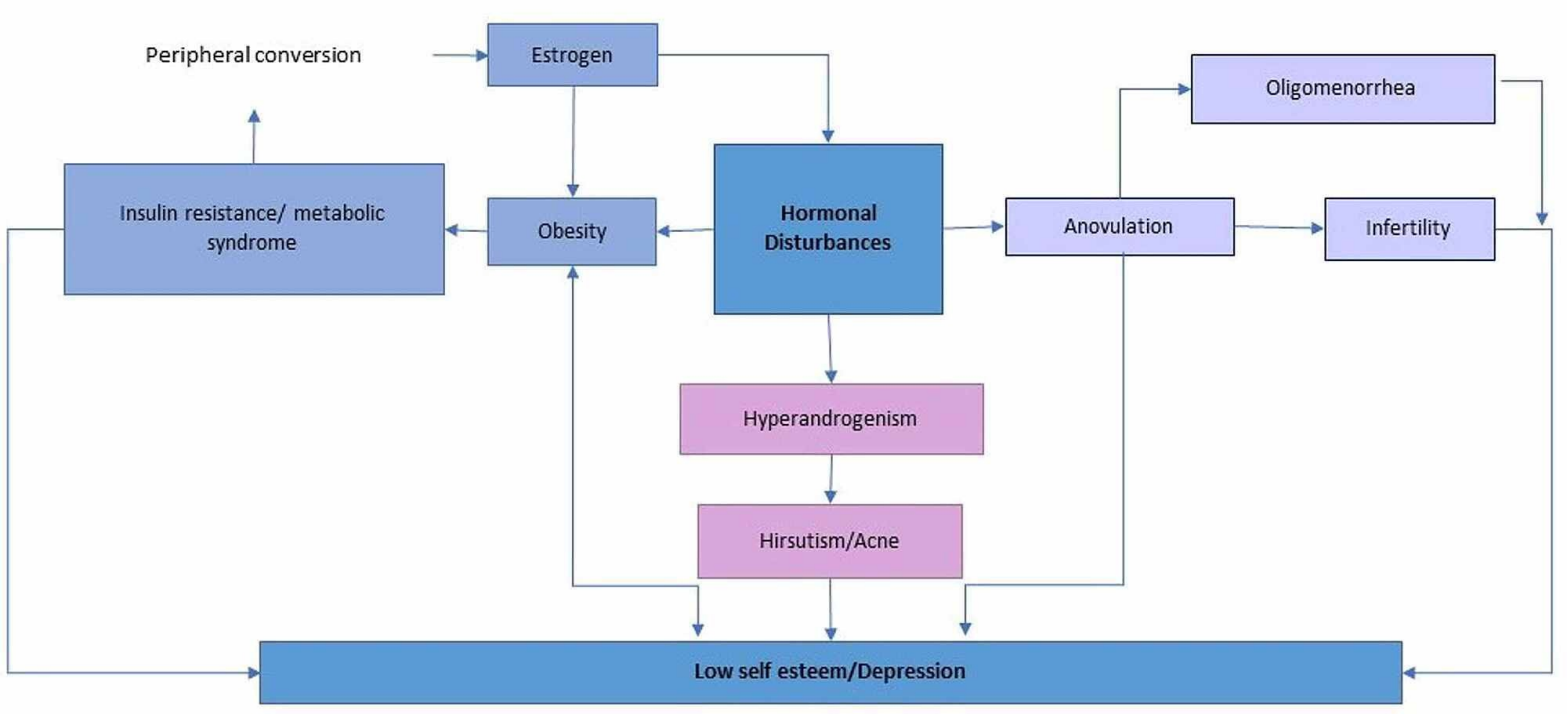
Multiple manifestations of PCOS linked to depression. PCOS: polycystic ovary syndrome

Therefore, considering the severe and disastrous outcomes of PCOS on the health of the affected women, it is crucial to understand the interplay between the various manifestations of PCOS (Figure 1) and its link to developing depression in this high-risk population. The goal of this paper is to explore the possible causes of depression in women with PCOS, as well as to highlight the gaps in our understanding of this relationship to guide further research.

The diagnostic criteria for PCOS have evolved throughout the years. However, fundamental to each diagnostic criteria lies a triad of menstrual irregularities, manifestation of hyperandrogenism, and ultrasound imaging showing polycystic ovaries [6]. The different diagnostic criteria are listed in Table 1.

**Table 1:** Different diagnostic criteria used for PCOS. Information from Azziz et al. (2016) [3]. PCOS: polycystic ovary syndrome

	1990 National Institutes of Health criteria	2003 Rotterdam criteria	2009 Androgen Excess and PCOS Society
	Must have both of the findings	Must have 2 out of the 3 findings	Must have 1 and either 2 or 3
Menstrual abnormalities: amenorrhea/oligomenorrhea	1	1	1
Hyperandrogenism: Biochemically or clinically (hirsutism/acne)	2	2	2
Ultrasound imaging: polycystic ovaries		3	3

## Review

Despite the surge of interest in the last 10 years to better understand the possible causes of depression in women with PCOS, the exact mechanism causing this higher disease burden is still unclear and thought to be multifactorial. This review explores these possible mechanisms.

Hypothalamic-pituitary-ovarian/adrenal axis and its dysregulation in polycystic ovary syndrome

**Figure 2:**
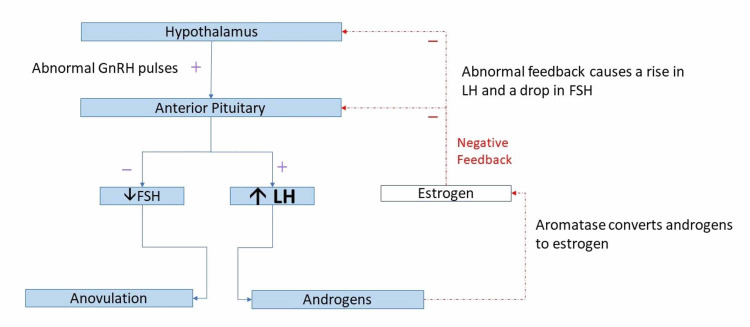
HPO axis disturbance in PCOS. GnRH: gonadotropin-releasing hormone; FSH: follicular stimulating hormone; LH: luteinizing hormone; HPO: hypothalamic-pituitary-ovarian; PCOS, polycystic ovary syndrome

**Figure 3:**
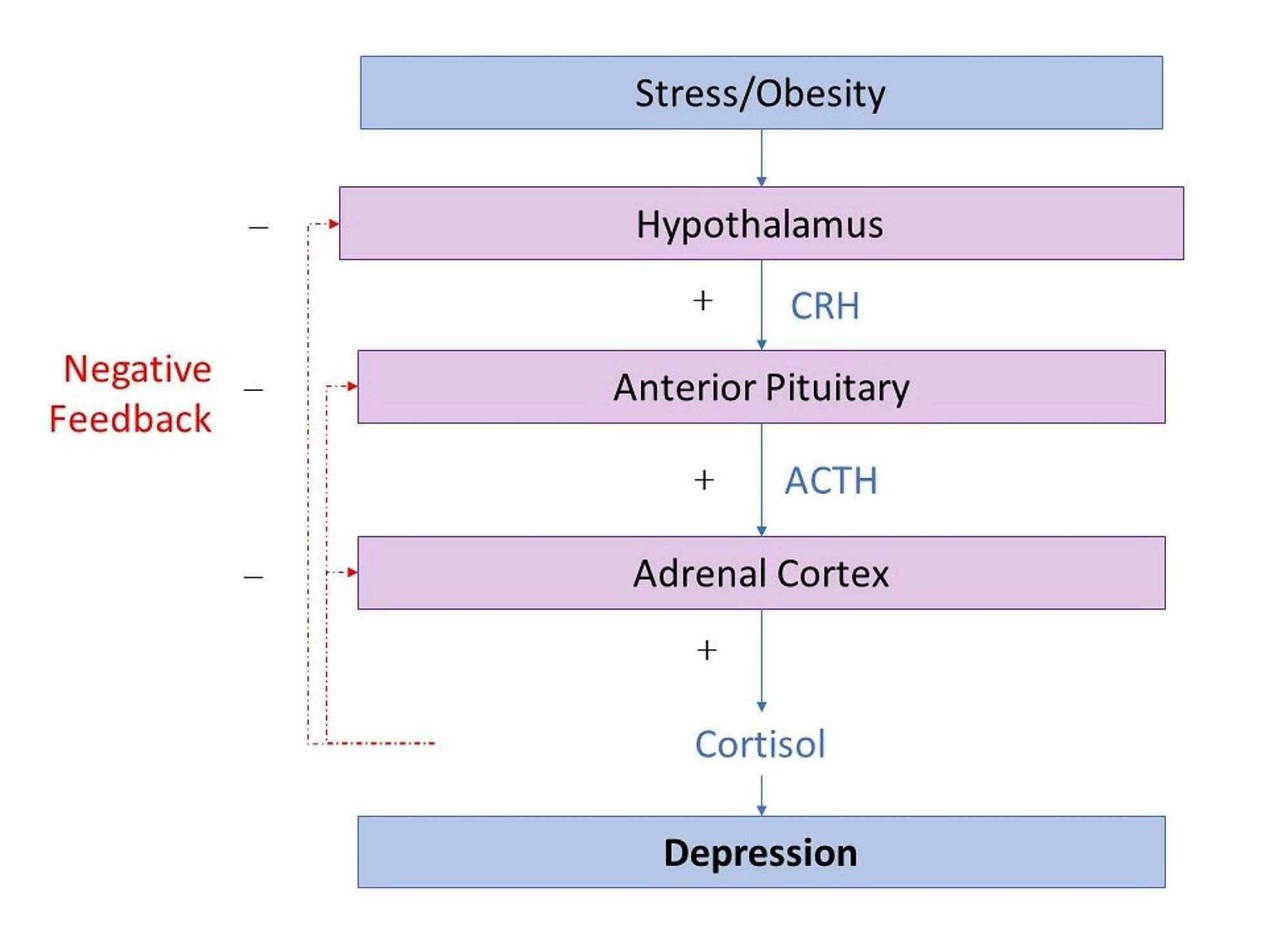
How stress and obesity disturbs HPA axis and its link to depression. CRH: corticotropin-releasing hormone; ACTH: adrenocorticotropic hormone; HPA: hypothalamic-pituitary-adrenal

The diagnosis of PCOS is based on an underlying abnormality in the feedback mechanisms in the hypothalamic-pituitary-ovarian (HPO) axis (Figure 2) [16]. Another postulated mechanism for causing depression is a dysregulation in the hypothalamic-pituitary-adrenal (HPA) axis (Figure 3) [16]. Any form of stress can alter these two pathways [16]. High levels of stress hormones such as corticotropin-releasing hormone and increased cortisol have been linked as a possible cause of depression in the general population [16]. Older studies have shown that women with PCOS have higher cortisol levels after a stressor compared to control groups [17,18], and that women with PCOS are at risk for higher levels of stress and anxiety [19].

However, no recent studies have confirmed this underlying mechanism as the cause of depression in women with PCOS. Studies have also used small sample sizes, and more extensive clinical trials should explore the alterations in all the hormones in the various pathways involved in the HPA and HPO axes, as well as their direct link to depressive symptoms in women with PCOS

Elevated androgens linked to depression

Hyperandrogenism can manifest clinically or biochemically and forms a part of the PCOS diagnostic criteria [20]. Its role in the causal link to depression is controversial; although there are many hypotheses, none are consistently reproducible.

Biochemical Hyperandrogenemia

Biochemical hyperandrogenemia is defined by elevated levels of androgens in the blood. It is confirmed through lab investigations to prove elevated levels of total testosterone, free testosterone, free androgen index (FAI), and dehydroepiandrosterone sulfate. There are many pitfalls to selecting biochemical tests as they may not all be elevated in all patients [20,21].

These biochemical markers may be more relevant in specific age groups. Two large longitudinal studies showed that older women with higher baseline testosterone and increased testosterone changes during the perimenopausal period are associated with higher depression scores [22,23].

To the best of our knowledge, no previous studies have monitored the biochemical features of PCOS in adolescents, comparing them to a similar age group to elicit hyperandrogenemia at the time of PCOS diagnosis as a possible cause of depression in younger females. The majority of studies were conducted in an adult population, which shows a possible gap in our understanding and early comprehensive management of the disease.

A meta-regression study concluded that there was no association between any measurement of testosterone and depression [20]. Small cross-sectional studies have shown a positive correlation between women with PCOS and a high FAI level, which increased the risk for mood disturbances [24,25]. However, based on this study design, no causal relationship can be determined. More extensive studies need to be done in multiple populations to determine a temporal relationship between hyperandrogenemia and depression in women with PCOS.

Clinical Hyperandrogenism

Clinically, excess androgens can manifest as hirsutism, acne, and alopecia [21]. It is the clinical manifestation of the combination of circulating androgen level, androgen level at the local site, and the hair follicles’ sensitivity to the circulating hormones. Thus, the degree of severity of hirsutism does not always correlate with the androgen levels in the blood [21]. Hirsutism is graded based on the hair growth’s location and severity using the Ferriman-Gallwey (FG) score. A score of eight or higher is part of the diagnostic criteria for PCOS [26,27].

A meta-analysis conducted in 2017 concluded that women with PCOS had higher FG scores than control groups [28]. The higher the patient-rated FG scores, the higher the depression scores [28]. Depression was associated with increased odds of hirsutism in six different studies [28]. A study revealed that patient-rated FG scores and not the clinician-rated FG scores were significantly associated with the risk of depression [5]. Demonstrating that hirsutism is perceived differently by women, and even relatively little hair growth can cause embarrassment, social withdrawal, and self-consciousness [29].

The skin manifestations of elevated androgens can cause a negative self-image, and it may play a role in the possible mechanism of depression in women with PCOS. The data suggest that the background population, its cultural norms, and women’s interpretation of these symptoms determine their impact on mood symptoms.

Metabolic pathway and its link to depression

In women with PCOS, the prevalence of metabolic syndrome is high. Metabolic syndrome includes central obesity, hypertension, insulin resistance, and atherogenic dyslipidemia. PCOS combined with metabolic syndrome can be associated with long-term complications such as cardiovascular disease, diabetes mellitus, sleep apnea, and depression. Depression can negatively affect the motivation needed to improve lifestyle habits, resulting in worsening symptoms and further metabolic decline [30].

In the general population, obesity and insulin resistance have been associated with an increased risk of depression [31]. This metabolic pathway can be a possible cause of depression in women with PCOS.

Obesity

Obesity prevalence is as high as 60% among women with PCOS, and it is identified as a possible etiology for depression in this population [10]. A meta-regression study showed that a higher BMI was associated with women with PCOS and depressive symptoms compared to the control group without these symptoms [20]. However, the strength of association between these variables was small, and when participants’ BMI was matched, women with PCOS still had higher odds of depressive symptoms [20]. Longitudinal studies have concluded that metabolic phenotype (obesity) is a strong risk factor for enduring depression in women with PCOS [32].

The data show that PCOS is an independent risk factor for developing depression and cannot be fully accounted for by obesity alone. Furthermore, depressed PCOS patients are more likely to be obese.

The mechanism by which obesity causes depression is complex. Weight gain activates the inflammatory pathways, and thus obesity is viewed as an inflammatory state. In the general population, there is a bidirectional relationship between inflammation and depression. Obesity causes dysregulation in the HPA axis (Figure 3), which is involved in developing depression [20,33]. Furthermore, an external appearance that deviates from societal views of beauty, in this case, obesity, can worsen depressive symptoms and create an altered sense of female identity [34].

The association between depression and obesity is well established; however, the underlying mechanism can be due to the internal biochemical changes, or it can be a response to external societal pressures.

Insulin Resistance

Up to 70% of women with PCOS have insulin resistance, and they have a higher prevalence of diabetes mellitus compared to healthy young women [35]. Studies have shown that insulin resistance is common in women with PCOS even when accounting for BMI [36]. These findings show that the underlying mechanism in developing insulin resistance is not solely due to obesity, and it may play a more significant role in the causal pathway in multiple clinical presentations of PCOS.

Multiple studies have also found a positive correlation between insulin resistance and depression in a wide variety of populations [37]. A large, multicenter randomized trial involving 738 women with PCOS showed insulin resistance as an independent risk factor for depression in this population [13]. Multiple trials also showed a positive correlation between insulin resistance and the risk of depression [9,38,39]

Few studies did not find a link between insulin resistance and depression in women with PCOS; however, this could be due to the small sample size used, the study designs, or using a self-reporting tool [40,41].

Although there is an established link between insulin resistance and depression, the causal relationship is more likely to be bidirectional. This means that although insulin resistance can cause depression through biological pathways, depression, in turn, can cause insulin resistance through behavioral patterns such as a sedentary lifestyle and poor dietary habits [20,33].

The biological pathway involved in developing PCOS through insulin resistance is thought to be due to elevated cortisol. This hormone, in turn, causes an increase in the sympathetic nervous system and inflammatory markers. Insulin resistance has also been shown to decrease brain serotonin [42,43]. A study conducted using functional magnetic resonance imaging showed that insulin resistance altered connectivity in the brain. Studies have also showed neuroimaging changes in the limbic system during an emotional task in women with PCOS. Interestingly, these changes normalized with the treatment for insulin resistance [44,45].

Hence, insulin resistance can be a possible cause of depression in women with PCOS, and alternatively, treating abnormal insulin levels can reduce the development of depression. Longitudinal studies need to be conducted to see if depression, independent of obesity and other comorbidities, can be prevented by controlling abnormal glucose levels.

Menstrual abnormalities linked to depression

Women with PCOS commonly suffer from menstrual abnormalities such as anovulation, amenorrhea, and oligomenorrhea [46]. Limited data have compared these experiences with psychological distress. However, two semi-structured interviews showed that abnormal menstrual cycles are associated with a decreased sense of feminine identity. Some women revealed that they were not real women due to the unpredictability of their menstrual periods [7,8]. However, a study in Germany that included 120 women with PCOS found that the type of menstrual abnormality (amenorrhea or oligomenorrhea) had no significant difference in the distress experienced [46].

The contradictory results could be due to societal beliefs and cultural influences. The studies did not account for race and religious beliefs.

External pressures can change the severity of how menstrual irregularities are perceived. They can be viewed as more problematic than they are and influence psychological well-being through this mechanism [47].

Infertility

**Figure 4:**
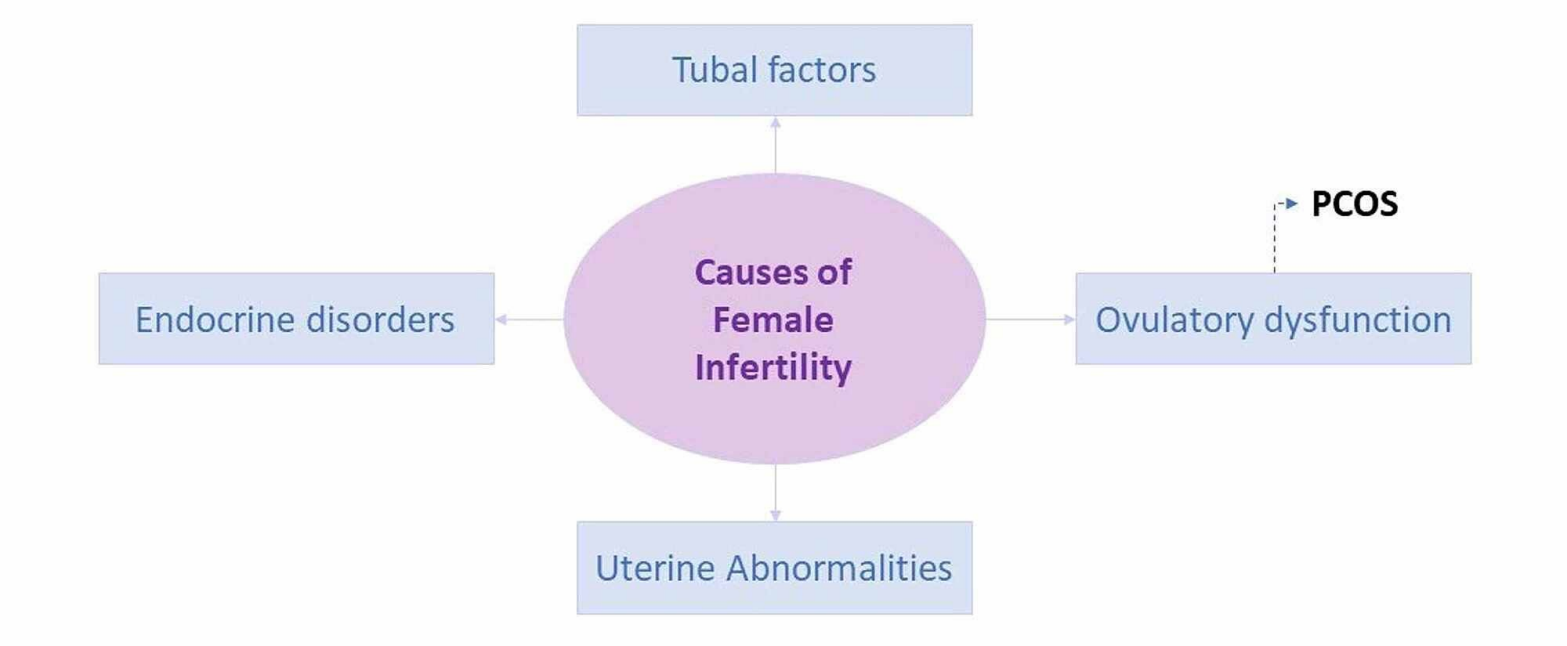
Causes of female infertility according to the World Health Organization. PCOS: polycystic ovary syndrome

Among the many causes of female infertility (Figure 4), ovulatory dysfunction due to PCOS comprises 75% [48]. The prevalence of depression in infertile people, despite the cause, ranges between 7% and 26% depending on the population [49]. Thus, infertility can be a contributory factor in developing depression. However, studies that included only infertile women or excluded all infertile women showed that depression scores were still higher in women with PCOS [5].

The data suggest that although infertility can be a contributory factor causing depression in women with PCOS, it cannot be solely due to infertility.

A few limitations were identified in this study. Some articles included were from only the abstracts, and as access to the full article was not achieved, specific examples may have been lost. Additionally, depressive symptoms and the impact on their quality of life are based on different subjective tools, which may cause recall bias in the studies chosen. As some of the studies included were cross-sectional in design, it is not possible to ascertain a causal relationship with depression.

## Conclusions

The underlying mechanisms leading to depression in women with PCOS were explored. There are most likely many interconnected and mutually reinforcing pathways that put these women at an increased risk for depression. The pathophysiology thought to play a role includes disturbances in the endocrine axes, hyperandrogenism and its clinical manifestations, alterations in the metabolic pathway, and menstrual abnormalities. The prevalence of depression in women with PCOS is high; thus, it is essential to understand the underlying mechanisms at play to address them effectively.

This paper adds to the understanding of the underlying cause of depression and can be used to adjust and improve guidelines to address this common and disastrous clinical complication. Depression adds to the global burden of disease, and if the mechanism is understood, preventative measures can be put in place for these high-risk women. Future research needs to focus on conducting long-term cohort studies and clinical trials to better understand the temporal relationships between the various manifestations of PCOS and its causal link to depression.
